# Ovariectomy shortens the life span of female mice

**DOI:** 10.18632/oncotarget.2984

**Published:** 2015-02-12

**Authors:** Valeria Benedusi, Elisa Martini, Marinos Kallikourdis, Alessandro Villa, Clara Meda, Adriana Maggi

**Affiliations:** ^1^ Center of Excellence on Neurodegenerative Diseases and Department of Pharmacological and Biomolecular Sciences, University of Milan, 20133, Milan, Italy; ^2^ Adaptive Immunity Laboratory, Humanitas Clinical and Research Center, 20089 Rozzano, Milan, Italy; ^3^ Department of Medical Biotechnologies and Translational Medicine, University of Milan, 20089 Rozzano, Milan, Italy

**Keywords:** female mammals, ovariectomy, life span, inflammation, estrogens

## Abstract

This study shows that lack of ovarian activity has a negative impact on the life span of female mice. The extent to which this phenomenon could be associated with the anti-inflammatory effect of estrogens was analyzed in metabolic organs and aorta, by quantitative analysis of mRNAs encoding proteins in the inflammatory cascade. We demonstrate that the TNFα, IL-1β, MCP-1, MIP-2 and IL-6 mRNA contents are increased in the liver, adipose tissue and aorta 7 months after ovariectomy (ovx) and this increased basal inflammation is maintained as the mice aged. In contrast, the extent of inflammatory gene expression is directly proportional to age in sham-operated mice. As a consequence, at 22 months, most of the inflammatory parameters examined were higher in the sham-operated group compared with the ovx group. These observations led us to propose that the decreased longevity of ovx mice may be due to an acceleration of the basal state of inflammation in metabolic organs, which is likely driven by the combination of a lack of estrogen-mediated anti-inflammatory activity and the loss of gonadal control of energy metabolism.

## INTRODUCTION

In an effort to limit the major societal burden of pathologies associated with human aging, recent research has focused on improving our understanding of the mechanisms regulating senescence and longevity. Cellular, animal and clinical studies led to a number of theories on the mechanistic underpinnings of aging [1–4]. Among those theories, the impairment of metabolic functions and deficits within the defense immune system are gaining increasing consideration [1, 5]. The metabolic and immune systems are closely associated [5], and a deregulated immune response may damage the activity of metabolic organs [6]. In contrast, unbalanced energy metabolism is a trigger for inflammatory processes [7]. Thus, the two systems may cooperate to create an unhealthy vicious cycle that accelerates senescence and decreases longevity.

A parsimonious use of energy prolongs life span by decreasing the probabilities of damage to the pathways controlling nutritional and internal energy deposits. In line with this view, reproduction, which ultimately requires energy, favors aging and reduces longevity [8]. Indeed, aging is inextricably linked to different aspects of reproduction in many species. For instance, the removal of germ cells is associated with a longer life-span in a large variety of oviparous, including worms, flies, insects, fishes and in male mammals [8–13]. In contrast, initial studies in female mammals (rats) indicated that ovx is associated with a shortened life span [14] and that the pathological deficiency or loss of ovarian function is associated with the derangement of energy metabolism and immune function (rodents and humans) [15]. These data suggest that the ovaries and their endocrine activity may have a positive effect on longevity in mammals. Supporting this theory, Nurse's Health study [16] reported that women had greater longevity after elective hysterectomy compared with those who received ovariectomy and hysterectomy. In addition, menopause is associated with metabolic dysfunction [15] and pathologies involving inflammation (e.g., osteoporosis and metabolic disorders, including diabetes, atherosclerosis, joint diseases and even neurodegeneration) [5, 17].

Estrogens regulate energy metabolism in several organs, including the liver, where these hormones strongly influence lipid and cholesterol metabolism [18, 19]. In fact, ovx in mice and menopause in women are associated with increased weight, decreased lean mass [20], visceral fat (vWAT) accumulation [21] and an increase of lipids in the liver. These changes may initiate a complex chain of events, where the formation of lipid deposits (e.g., in hepatocytes and adipocytes) induces macrophage recruitment and activation [6], resulting in an inflammatory state that intensifies metabolic dysfunctions and lipid deposition and worsens the inflammatory status [22, 23]. In addition, estrogens are anti-inflammatory molecules [24–26]. Thus, considering that generalized low-grade inflammation is a major factor in determining senescence [5], the loss of ovarian estrogens in aging females may aggravate the senescence process via an increase in generalized inflammation.

The aim of this study was to investigate the extent to which the loss of ovarian activity affects the production of proinflammatory mediators during aging. Because of the synergic, negative interplay between inflammation and energy metabolism, our study initially focused on two major metabolic organs: the liver and adipose tissue. We also investigated the aorta as a primary target of alterations in lipid metabolism and inflammatory processes. Our results indicate that ovx carried out in adult mice accelerates the accumulation of a number of products in the inflammatory cascade that are characteristic of aging in the liver, adipocytes and aorta. Importantly, ovx was associated with a significantly decrease in life span, suggesting that the ovx-dependent acceleration of aging was dependent on increased basal inflammation and may have detrimental consequences for the health of female mice.

## RESULTS

### Survival after ovariectomy

**Figure 1:**
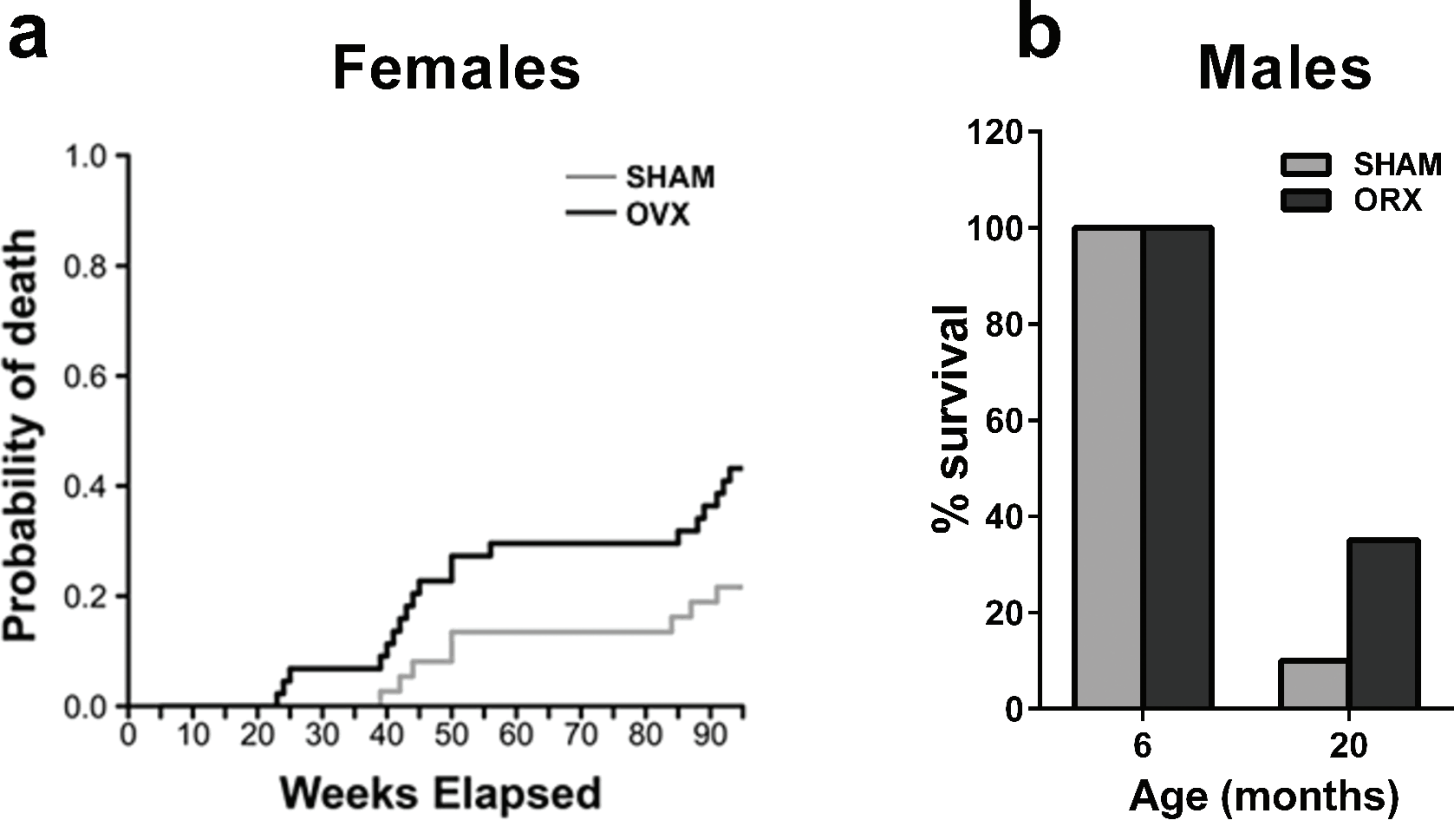
Gonadectomy and life span in female and male mice **(a)** Female PPRE-lucRepTOP™ were gonadectomized (OVX, *n* = 40) or sham-operated (SHAM, *n* = 40) at 5 months of age. Each group was randomly subdivided into 4 clusters of 10 animals each to be euthanized at 6, 12, 18 and 22 month of age to evaluate the state of inflammation. Predicted cumulative incidence curves for death by natural causes in SHAM and OVX animals were calculated as described in the methodology section. The risk of death by natural causes was significantly higher in ovx mice compared with sham-operated mice (*p* < 0.05). **(b)** Male ERE-LucRepTOP™ in the C57BL/6J background were orchiectomized (*n* = 40) or sham operated (*n* = 40) at 5 month of age. 20 animals from each group were euthanized at 6 months and 20 months, respectively. Data are expressed as the mean % survival ± SEM.

To evaluate the effect of ovx on inflammation, female mice were subjected to sham-operation or ovx at 5 months of age. The age of surgery was selected to assess the effect of ovary loss in mature, adult mice. The animals (10 per experimental group) were generated in 10 harems, randomized at the beginning of the experiment and exposed to the same environmental conditions throughout the study, as surgery and aging were carried out in our departmental facilities. Periodic vaginal smears (every 3 weeks) indicated that the sham-operated mice were cycling up to approximately 18 months. By 22 months, the cycles ceased, and the production of ovarian hormones was decreased, as indicated by uterus weight (Supplementary Figure 1). Competitive risk analysis for death (Figure 1a) demonstrated that the probability of death was significantly higher in the ovx group compared with the age-matched, sham-operated controls. This phenomenon was sex-specific because the survival rate of male mice orchiectomized (orx) at 5 months was 35% compared with 10% in the sham-operated mice at 20 months (Figure 1b). To evaluate the reproducibility of the effect of ovx on female survival, we analyzed the results of another study carried out using similar experimental conditions to those described in the Figure 1a, except the mice had slightly different genetic backgrounds and were maintained in a commercial breeding facility (see Experimental Procedures section). This study also indicated that ovx mice had a higher probability of death than sham-operated mice (*P* = 0.005 with Grey's test) (data not shown). Therefore, gonadectomy had a positive effect on male, but not female, mouse longevity.

### Ovx accelerates the inflammatory process associated with aging

**Figure 2:**
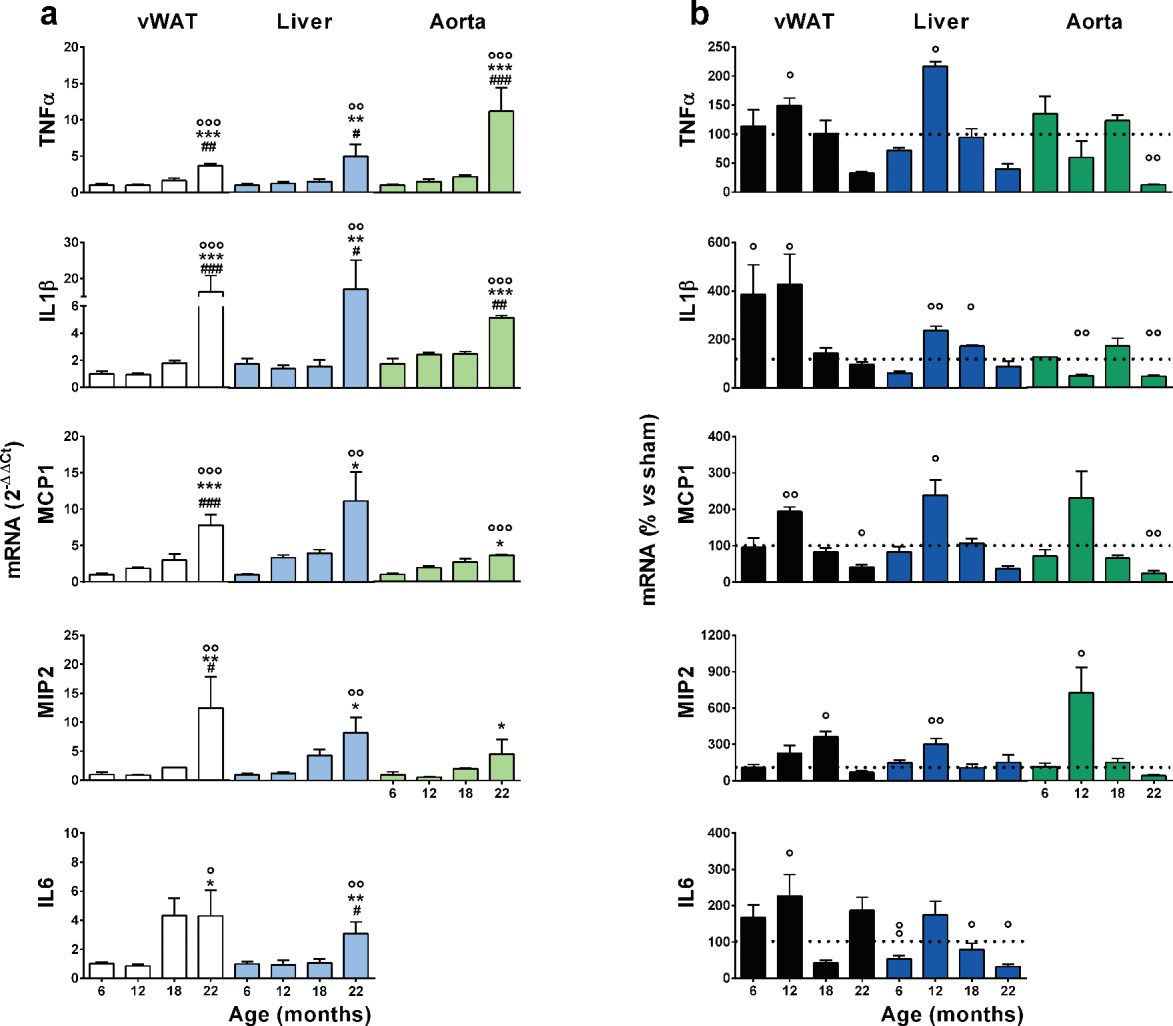
Effects of aging and ovariectomy on the basal state of inflammation in metabolic organs and the aorta **(a)** Tissues from sham operated mice collected in Figure 1 were stored until rtPCR analysis. Data are expressed as the mean ± SEM and were analyzed by one-way ANOVA, followed by Bonferroni *post hoc* test. °*P* < 0.05; °°*P* < 0.01, °°°*P* < 0.001 *vs*. 6 months; *P < 0.05. **P < 0.01, ***P < 0.001 *vs*. 12 months. ^#^*P* < 0.05; ^##^*P* < 0.01, ^###^*P* < 0.001 *vs*. 18 months. **(b)** Data represent the percent change of ovx *vs.* sham operated mice. Statistical significance was determined by comparing the raw numbers of ovx and sham-operated mice using unpaired *t*-tests. °*P* < 0.05; °°*P* < 0.01, °°°*P* < 0.001 ovx *vs*. sham. The experiment was repeated with a second cohort of aged ovx and sham-operated mice with superimposable results.

Increased basal inflammation is strictly associated with senescence [5]. Thus, we measured mRNAs encoding proinflammatory cytokines and chemokines in 2 major metabolic organs, the liver and white adipose tissue (perigonadal and abdominal, vWAT) [5]. We also studied the aorta as a primary target of alterations in lipid metabolism and inflammatory processes in female aging [27]. All cycling mice were euthanized at metestrus to prevent variability in the expression of inflammatory genes due to fluctuating levels of circulating sex hormones in the reproductive cycle. The mRNAs encoding tumor necrosis factor-α (TNFα), interleukin-1β (IL1β), macrophage chemoattractant protein-1 (MCP-1), macrophage inflammatory protein-2 (MIP-2) and interleukin-6 (IL-6) were measured by quantitative rtPCR in tissue extracts. In sham-operated mice, the mRNAs encoding these pro-inflammatory mediators increased steadily with age, and at 22 months, the mRNAs investigated were significantly higher than at 6 months (Figure 2a). In the ovx mice, the effect of age was not as obvious; the accumulation of inflammatory precursors was higher than in the sham-operated group at 12 months, and the relatively high expression of these mRNAs did not increase with aging (in some cases they diminished, e.g., MCP-1) (Supplementary Figure 2). The effect of ovx was significant when we expressed the level of each inflammatory mediator as a percent of the respective sham-operated age group (Figure 2b); generally, the amount of inflammatory mRNA accumulated in the ovx mice was significantly higher than that in the sham-operated mice at approximately 12 months of age, and with few exceptions, the content of inflammatory products in the tissues of ovx mice was significantly lower in the sham-operated group than in the ovx group by 22 months. Thus, ovx accelerated the age-associated accumulation of inflammatory molecules. Interestingly, this effect required several months (6–7) to become significant, suggesting that the mere absence of the anti-inflammatory action of circulating estrogens was not sufficient to impair the homeostasis of the inflammatory system. These data led us to hypothesize that the effect of ovx was initially minor, but became significant when associated with the functional alterations of the immune system that occur with aging.

### The effect of short- and long-term ovariectomy on inflammation in adipocytes

**Figure 3:**
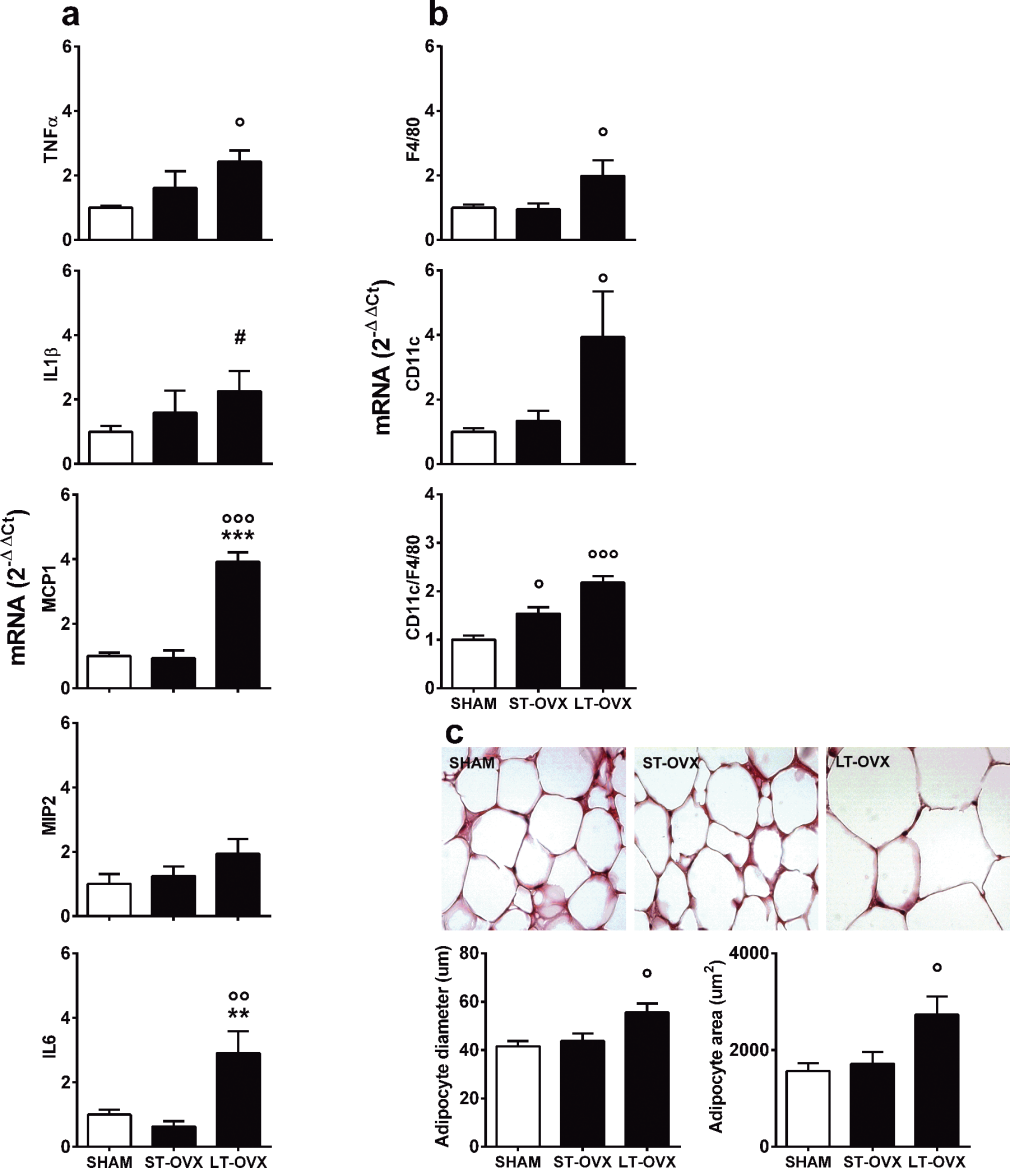
Effect of short- and long-term ovx in vWAT Sham-operated female C57BL/6J mice at metestrus (*n* = 8) and 1 month (ST-OVX; *n* = 8) or 4 months (LT-OVX; *n* = 8) after ovx were euthanized at 5 months of age, and vWAT was rapidly removed and snap frozen for further analysis. **(a)** rtPCR was used to measure cytokine and chemokine mRNA content in vWAT. **(b)** Expression of markers for macrophages (F4/80) or macrophage M1 polarization (CD11c) were measured by rtPCR in vWAT. **(c)** *Top panel*: Representative images of H&E staining of vWAT tissues from sham-operated, ST-OVX and LT-OVX mice. *Bottom panel*: graphs represent the quantification of adipocyte size obtained using the Adiposoft ImageJ software. Data are expressed as the mean ± SEM and were analyzed by one-way ANOVA, followed by Bonferroni's *post hoc* test. °*P* < 0.05; °°*P* < 0.01; °°°*P* < 0.001 *vs*. sham-operated. *P < 0.05; **P < 0.01; ***P < 0.001 *vs*. ST-OVX. ^#^*P* < 0.05 *vs*. sham-operated, as determined by unpaired *t*-test.

To test the hypothesis above, we investigated the effect of ovx in very young animals. Surgery (sham or ovx) was performed at 1 month, immediately after the first estrous cycle, (long-term ovx, LT-OVX) or 4 months (short-term ovx, ST-OVX), and all mice were euthanized at 5 months. TNFα, IL1β, MCP-1, MIP-2 and IL-6 mRNAs were measured in the vWAT using rtPCR. Figure 3a shows that LT-OVX, but not ST-OVX, significantly augmented TNFα, IL1β, MCP-1 and IL-6 compared with the sham-operated counterpart. Remarkably, TNFα mRNA only was significantly augmented by LT-OVX in the liver (Supplementary Figure 3). These results led us to hypothesize that, at least in vWAT, the delayed effect of ovx on the accumulation of inflammatory molecules was the result of a mild activation of the native immune system due to decreased circulating estrogens; this subtle immune deficiency required months to manifest. The lack of marked, significant inflammation in the liver suggested that, in younger mice, this organ could cope better than vWAT with lower levels of circulating sex hormones. To further investigate the consequences of ovx in vWAT, we measured the markers, F4/80 (as a general marker of macrophages) and CD11c (indicator of M1 state of polarization), in ST-OVX and LT-OVX [28–30]. Figure 3b illustrates that both of these markers were significantly increased in the LT-OVX group. However, when we analyzed the percentage of activated macrophages (CD11c versus F4/80 mRNA), we also observed a significant change in the ST-OVX group, suggesting that ovx was associated with a rapid change in macrophage activation and caused macrophage recruitment in vWAT. The deficiency of circulating estrogens might favor the slow accumulation of inflammatory products. In LT-OVX mice vWAT, we observed a significant enlargement of adipocytes (Figure 3c), suggesting that ovx was also associated with impaired adipocyte homeostasis.

**Figure 4:**
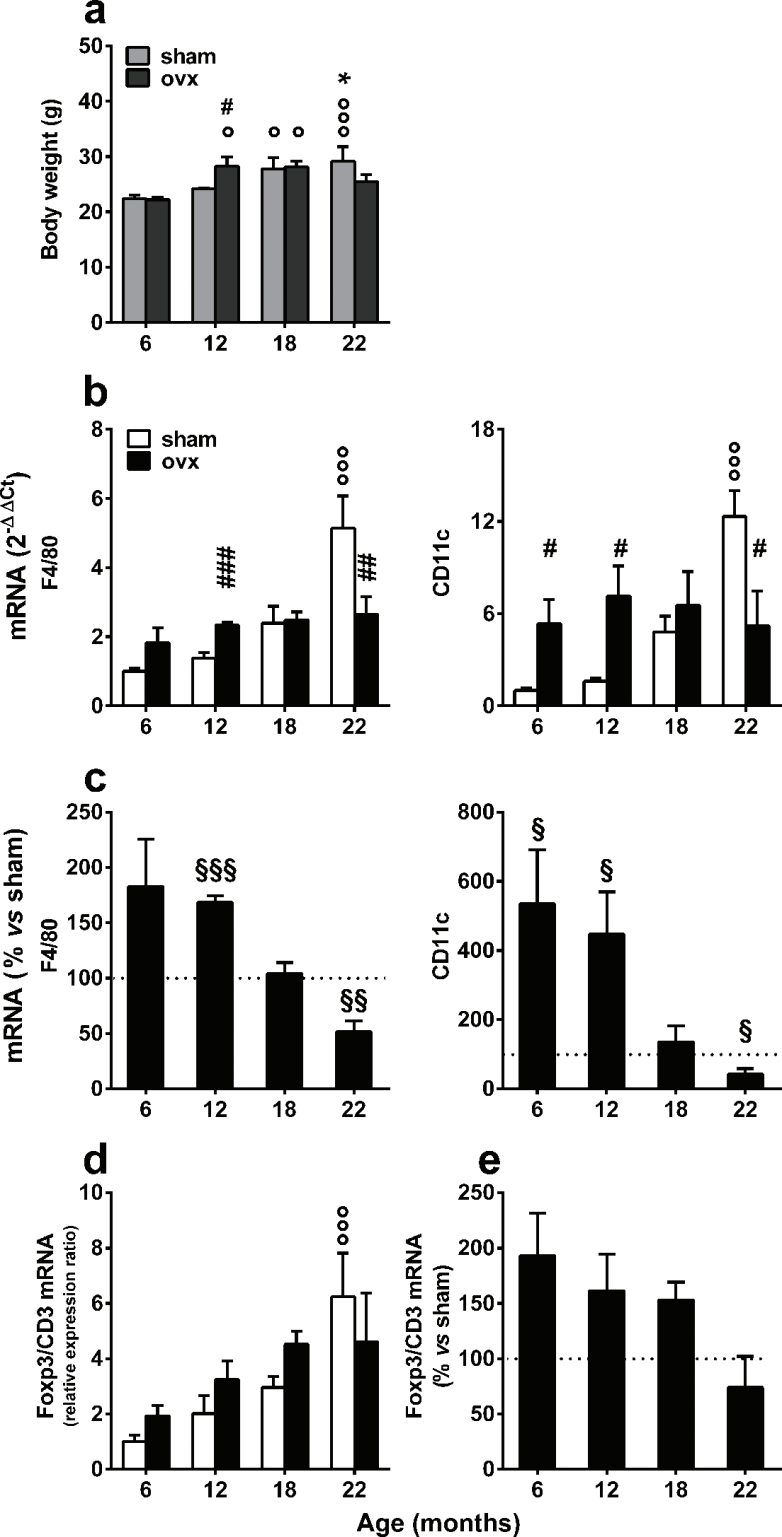
Expression of immune response markers in the vWAT of sham-operated and ovx aging mice **(a)** The body weight of each mouse was measured before euthanasia. Data were analyzed by two-way ANOVA, followed by Bonferroni's *post hoc* test. °*P* < 0.05; °°*P* < 0.01; and °°°*P* < 0.001 *vs*. 6 months of age; *P < 0.05 *vs*. 12 months of age; ^#^*P* < 0.05 *vs*. age-matched sham operated. **(b)** F4/80 and CD11c mRNA contents were measured using rtPCR of tissue extracts from the vWAT of sham-operated and ovx female mice. °°°*P* < 0.001 *vs.* 6 months of age; ^#^*P* < 0.05; ^##^*P* < 0.01; and ^###^*P* < 0.001 ovx *vs*. sham. Data were analyzed by two-way ANOVA, followed by Bonferroni's *post hoc* test. **(c)** Macrophage activation in vWAT was expressed as the ratio of CD11c to F4/80 mRNA content. Statistical analysis was performed by comparing the raw numbers from ovx and sham-operated mice by two-way ANOVA, followed by Bonferroni's *post hoc* test. §*P* < 0.05; §§*P* < 0.01; and §§§*P* < 0.001. **(d)** The F4/80 and CD11c mRNA content of ovx mice are shown as the percentage of the mean of the sham-operated mice of the same age. Data were analyzed by two-way ANOVA, followed by Bonferroni's *post hoc* test. °°°*P* < 0.001 *vs*. 6 months of age. **(e)** Treg content in vWAT was expressed as the ratio of Foxp3 to CD3 mRNA levels. The Foxp3/CD3 ratio of ovx mice was represented as a percentage of the mean of sham-operated mice of the same age. Data were analyzed by two-way ANOVA, followed by Bonferroni's *post hoc* test. No statistical significance was found. All data are expressed as the mean ± SEM. Analysis of markers of immunity and body weight was repeated in separate aging experiments with superimposable results.

These alterations of fat metabolism in association with ovx were predictable, as previous studies have demonstrated that liver lipid metabolism is highly modified by the loss of ovarian functions [19]. Furthermore, we found that ovx affected animal weight. Figure 4a demonstrates that the ovx mice weighed significantly more than did the sham-operated at 12 months, but their weight did not continue to increase with age. In contrast, weight increased progressively in sham-operated mice. A quantitative analysis of macrophage markers revealed that there was a continuous, progressive increase in both F4/80 and CD11c in sham-operated animals, and this increase became significant at 22 months. In contrast, the expression of these markers was significantly higher in the ovx group at 6–12 months of age compared with the sham-operated group, but at 22 months, it was lower than the age-matched controls. Interestingly, CD11c expression was increased in the 6-month group at one month after ovx; this was not observed in younger animals, suggesting an accrued sensitivity to an inflammatory stimulus (ovx) at 5 months of age (Figure 4b). The comparative analysis of the expression of F4/80 and CD11c in sham and ovx mice clearly indicated that ovx mice have an accelerated inflammatory response that fades over time (Figure 4c).

### Is ovx affecting FoxP3+ regulatory T cell accumulation in vWAT?

To evaluate whether ovx affected other functions of the immune response, we investigated the response of Foxp3 T regulatory cells (Treg). Tregs are a) important guardians of the immune response and highly involved in the regulation of the metabolic consequences of inflammation [31] and b) modulated by estrogens [32]. Foxp3 mRNA was measured by rtPCR, and we used the lineage-specific marker, Foxp3, and a pan-marker gene for T cells, CD3, to determine the proportion of the Treg cell sub-population. Similar to the ovx group, Foxp3 mRNA increased during the course of aging in vWAT (Figure 4d); however, when we compared the Foxp3 expression in the two groups, the content of Treg mRNA in ovx mice was progressively lower than in sham-operated counterparts with aging, indicating an incremental impairment in the ability of the immune system to suppress the inflammatory response associated with aging and ovx. Several studies demonstrated that Foxp3+ Treg cells might be derived from the recruitment of circulating Treg cells and through the conversion of resident T cells or *in loco* clonal expansion in vWAT and non-lymphoid tissues. Therefore, we verified whether aging and ovx affected the amount of Foxp3+ Treg cells in the spleen, a lymphoid organ rich in these cells. The data in Supplementary Figure 4 demonstrate that Foxp3/CD3 mRNA did not increase with aging in the spleens of either sham or ovx mice, indicating that these variations occur selectively in vWAT.

## DISCUSSION

The present study indicates that removing the gonads in adult female mice is associated with decreased longevity and acceleration of the age-dependent progression of basal inflammation.

### Ovariectomy accelerates the age-dependent progression of basal inflammation

The negative effect of ovx on life span in female mammals is well documented [14, 16], but the mechanisms involved in this process are poorly understood. Here, we report that ovx accelerates age-dependent progression of basal inflammation. The extent to which ovx affects the immune system either directly, by diminishing the efficiency of the inflammatory processes, or indirectly, by altering energy homeostasis and inducing a metabolically driven, chronic, low-grade inflammation that plays a crucial role in aging, remains unknown.

Estrogens are natural anti-inflammatory agents [24–26], and the lack of ovarian-mediated production of these sex hormones leads to the excessive accumulation of inflammatory molecules. Ovx is also associated with the rapid alteration of lipid metabolism and increased lipid deposits in the liver [19]. The accumulation of fat triggers the following physiological effects: *i.)* increased mitochondrial β-oxidation and the production of reactive oxygen species with insufficient suppression of lipolysis and *ii.)* the recruitment and activation of macrophages that initiate the secretion of proinflammatory factors in both fat and the liver. In lean individuals, small adipocytes promote metabolic homeostasis, whereas enlarged adipocytes recruit macrophages [6], promote inflammation [33] and release a range of factors that predispose toward metabolic diseases in obese individuals (e.g., insulin resistance) [34]. Studies comparing the responses of male and female mice to obesity and high-fat diet indicated that there was less immune cell infiltration and oxidative stress in the adipose tissue of females [35], indicating that high levels of circulating estrogens represent a protective factor against the inflammatory reactions induced by the stress pathways involved in energy metabolism.

The fact that an augmented inflammatory status is observed at 6–7 months after ovx did not allow us to draw definitive conclusions regarding the main target of estrogen that provides its beneficial effect, but it is possible to envisage a scenario in which *a*.) the alteration of energy homeostasis requires time to significantly affect energy deposits and trigger low-grade inflammation and *b*.) the lack of estrogens impairs macrophage activity, which significantly increases the accumulation of inflammatory molecules.

CD11c expression was increased relative to F4/80 1 month after ovx, suggesting that macrophages are rapidly activated in the vWAT after ovx. These data indicate that fat macrophages play a role in the increased inflammation observed at later times after ovx. The involvement of the Treg system in quenching the immune reaction does not appear to be a key element for suppressing the response to ovx. Indeed, as soon as 1 month after ovx, Treg cells are enriched in the T cell population in fat, but this response is not sufficient to counteract macrophage activation. Furthermore, the number of Foxp3 cells appears to decrease with time after ovx. In contrast, we observed that the number of Foxp3+ cells increased with aging in mice bearing intact ovaries, indicating that this system continuously attempts to limit the extent of inflammation induced by the senescent organism.

Several studies have described the effects of aging on circulating cytokines and demonstrated that inflammatory markers are significant predictors of mortality in old humans [7], but the extent to which inflammation is a cause or a consequence of aging remains unclear. However, only a handful of publications have investigated the modulation of cytokine production in metabolic organs, such as the vWAT and the liver, during physiological aging, and even fewer studies have investigated the effects of female aging and the lack of ovarian hormones on basal inflammation in these organs. This study fills this knowledge gap by demonstrating that ovx accelerates basal inflammation and shortens the life span. We propose that the ovx-induced acceleration of generalized inflammation represent the cause, or simply an indicator, of early damage precipitating the senescence process that, once induced, progresses with time, independently of the inflammatory system; at 22 months, ovx mice had lower basal inflammation than did sham mice, but the former's survival was significantly reduced.

### Reproduction and longevity are not negatively correlated

Our observation that removing the gonads in adult female mice decreases longevity contradicts previous investigations, suggesting that a trade-off exists between the activity of the reproductive system and longevity, whereby the lack of reproduction or the ablation of reproductive organs is associated with a longer life span [36]. Most prior studies on this subject utilized oviparous species and male mammals. Confirming previous studies, orx was not associated with diminished life span in males in our study, but studying the aging of female mice with identical genetic backgrounds that were reared in the same environmental condition revealed a sex-specific effect of gonadal ablation in mammals.

In oviparous, all of the nutrients stored in the egg that are essential for the development and growth of the embryo are produced by metabolic organs (primarily by the liver and, in less evolved animals, organs with hepatic functions) upon gonadal stimulation. This reciprocal control between gonads and the liver represents a simple, but extremely efficacious, means to prevent the unnecessary synthesis of energy molecules during gonadal malfunction and the blockage of reproductive functions when the energy supply is low.

In mammals, the production of a fertile egg represents only the initial step in reproduction. Once fertilized, the egg requires a growing supply of energy from the mother to ensure its development as an embryo and its maturation after birth. It is foreseeable that this change in the reproductive strategy caused a major revision in the liver-gonads dualistic control at each stage of fertility (puberty, pregnancy, lactation, and phases of the reproductive cycle), which require very different metabolic activities. Thus, evolution must have selected females in which the mechanism governing energy homeostasis had the flexibility necessary to adapt to these highly mutable requirements. Therefore, it is conceivable that with placentation, female metabolism diverged significantly from the ancestral system. Of course, mammalian males were not affected by these changes and maintained the ancestral mechanisms controlling energy metabolism; this may explain why there is a significant dimorphism in the physiology of energy metabolism in mammals and why the inverse relationship between longevity and reproduction was maintained from nematodes to humans in males, but not females.

The hypothesis that gonadal hormones have acquired significant control over the activities of metabolic organs in female mammals is rooted in significant experimental evidence demonstrating that estrogens have a major influence on glucose [37] and lipid [19] metabolism and clinical data showing that pathological and age-related ovarian dysfunction are closely associated with alterations in energy metabolism. Indeed, estrogens appear to play a key function in the signaling between reproductive and metabolic systems in mammals, including humans [15, 19, 38]; experimental and clinical evidence indicates that the loss of ovarian function in women at menopause is associated with the onset of an atherogenic lipid profile [39], which increases the risk of cardiovascular disease [40, 41] and a number of pathologies characterized by a significant inflammatory component.

## METHODS

### Animals

All mice used were bred on the C57BL/6 background. For the aging experiments, we used transgenic strains engineered to follow via *in vivo* imaging the activity of intracellular receptors. In the experiment shown in Figure 1a, PPRE-lucRepTOP™ (TOP srl, Lodi, Milan, Italy) in C57BL/6J was obtained from the original C57Bl/6xDBA/2 F2 through over 21 backcrossings. The males shown in Figure 1b and the females used to repeat the aging experiment contained ERE-LucRepTOP™ (TOP srl, Lodi, Milan, Italy) in C57BL/6J background.

To limit any genetic, environmental or rearing effects, all mice were born during the same period of time by trio breeding (one male and two females). All littermates were randomly distributed among the different experimental groups. The size of each experimental group was dictated by the sensibility of our assays. For aging experiments, each experimental group was composed of 10 mice, and the groups contained 8 mice for all other experiments. Mice were bred in our Departmental facilities and at Harlan commercial facilities (Corezzana, Italy). Mice were housed in ventilated plastic cages (335 cm^2^ floor area and 13 cm height) with hardwood chip bedding, and the animals were provided filtered water *ad libitum*. The number of animals/cage never exceeded 4. The animal room was maintained within a temperature range of 22–25°C, with an automatic 12 h light-dark cycle (lights on at 0700 h). The mice were maintained on a standard, estrogen-free diet (Mucedola 4RF21) (https://www.google.it/?gws_rd=ssl#hl=it&q=Mucedola+4RF21) and were moved to a certified estrogen-free diet 1 week before the experiment (AIN93M, Mucedola, Settimo Milanese, Italy) (http://www.zeiglerfeed.com/product_literature/lab%20research%20literature_Research/Purified%20Rodent%20Diet%20AIN-93M.pdf).

For ovx, the mice were anesthetized with a 50 μl s.c. injection of a ketamine (93.6 mg/kg, Ketavet 100; Intervet, Milan, Italy) and xylazine (7.2 mg/kg, Rompun; Bayer, Milan, Italy) solution and were then subjected to ovx or sham surgery. In the aging experiment, mice were subjected to ovx at 5 months of age, and in the experiment reported in Figure 3, mice were subjected to ovx at 1 month, after the first estrous cycle. As reported in the literature [42], vaginal opening occurs at day 28.1± 2.0 in wild type C57Bl/6 mice, when they weigh an average of 14.6 grams. Trained technicians performed the surgeries, and we achieved 100% success, as demonstrated by both vaginal smears and uterus weight at the end of the experimentation.

At the indicated time points (6–12–18–22 months for the aging experiment and 5 months for the ST/LT OVX experiment), mice were anesthetized using ketamine (93.6 mg/kg, Ketavet 100; Intervet, Milan, Italy) and xylazine (7.2 mg/kg, Rompun; Bayer, Milan, Italy) and euthanized by cervical dislocation. The liver, vWAT and aorta were quickly excised, snap frozen in dry ice and stored at −80°C for subsequent gene expression analysis. Portions of the vWAT (from the experiment in Figure 3) were fixed in 4% paraformaldehyde for 24 h and then embedded in paraffin. Sham-operated mice were all euthanized in the same phase of the estrous cycle, metestrus.

During the reproductive cycle of the sham-operated mice, the phase of the cycle was assessed by blind analysis of dry mount of vaginal smears stained using the May-Grünwald-Giemsa method (MGG Quick Stain Kit; Bio-Optica, Milan, Italy), according to the manufacturer's protocol. During the aging experiment, smears were collected for one week at three-week intervals. Mice were defined as cycling when at least a proestrus was observed during a 1-week period.

### Ethics statement

Investigation has been conducted in accordance with the ethical standards and according to the Declaration of Helsinki and according to the Guide for the Care and Use of Laboratory Animals, as adopted and promulgated by the US National Institute of Health, and in accordance with the European Guidelines for Animal Care and Use of Experimental Animals. In addition, the experiments were approved by the Italian Ministry of Research and University and controlled by a panel of experts at the Department of Pharmacological and Biomolecular Sciences (University of Milan, Milan, Italy).

### Real-time PCR gene expression analysis

The vWAT, liver and aorta were homogenized in TRIzol^®^ (Life Technologies, Carlsbad, CA), (6% w/v vWAT and liver, and 16% w/v aorta) using a TissueLyser (QIAGEN, Milan, Italy), and RNA was purified using the RNeasy MiniKit (QIAGEN, Milan, Italy), according to the manufacturer's instructions. To prepare the cDNA, 1 μg of RNA was denatured at 75°C for 5 min in the presence of 1.5 μg of random primers in a 15 μl final volume. Deoxynucleotide triphosphate and Moloney murine leukemia virus reverse transcriptase were added at final concentrations of 0.5 mM and 8 U/μl, respectively, in a final volume of 25 μl. The RT reaction was performed at 37°C for 1 h, and the enzyme was inactivated at 75°C for 5 min. Control reactions without the addition of the RT enzyme were performed using 20% of the samples.

The RT-PCR experiments were performed using TaqMan technology and TaqMan Gene Expression Assays (Life Technologies, Carlsbad, CA, USA) to analyze mouse TNFα (Mm00443258_m1), IL-1β (Mm00434228_m1), macrophage chemoattractant protein-1 (MCP1; Mm00441242_m1), macrophage inflammatory protein-2 (MIP2; Mm00436450_m1), IL-6 (Mm00446190_m1), F4/80 (Mm00802529_m1), CD11c (Mm00498698_m1), CD3 (Mm005996484_g1) and Foxp3 (Mm00475162_g1). We used the 18SrRNAVIC-MGB PDAR (Life Technologies, Carlsbad, CA, USA) as a reference for all of the assays.

The reactions were carried out according to the manufacturer's protocol using a 7900HT fast real-time PCR system (Applied Biosystems Inc., Foster City, CA, USA), and the data were analyzed by using the 2^−ΔΔCt^ method [43].

For Foxp3 expression analysis, tissues were homogenized in 1 ml of PureZol RNA isolation reagent (Bio-Rad, Hercules, CA, USA), according to the manufacturer's instructions, using GentleMACS and GentleMACS M Tubes (Miltenyi Biotec GmbH, Bergisch Gladbach, Germany). Retrotranscription was performed using a High Capacity cDNA Reverse Transcription kit (Applied Biosystems Inc., Foster City, CA, USA). Relative gene expression was calculated using Rn18s (Mm03928990_g1) as an endogenous control, and all samples were normalized to the mean relative mRNA expression of the sham group.

### Determination of adipocyte size

Five-micrometer-thick sections of paraffin-embedded vWAT were stained with Mayer's Hematoxylin and Eosin (H&E). Images of the staining were captured at X2000 magnification using a Zeiss Axioscope microscope equipped with a digital camera (Carl Zeiss, Thornwood, NY). Adipocyte area and average diameter were determined using Adiposoft, a software program that utilizes the NIH's open-share Fiji program (http://sw.wikkii.com/wiki/Adiposoft). Macros were developed to determine which cells to include in the size calculations (they had to be above a certain level of circularity and size to exclude pre-adipocytes or other cell types), and the area and Feret's diameter were calculated for each adipocyte assigned to each animal. For each mouse, 25–35 adipocytes from three separate sections were analyzed to determine the mean adipocyte volume.

### Statistical analysis

Unless otherwise stated, statistical significance was assessed by ANOVA using Bonferroni's multiple comparison *post hoc* test, using GraphPad Prism 5 (GraphPad Software, San Diego, CA).

For aging experiments, the mice were sacrificed at 6 month interval, so death from “natural causes”, which is the focus of our survival analysis, could not be observed for all mice. The death due to sacrifice of mice represents a competitor to death from natural causes; thus, these parameters were analyzed using methods appropriate for the competing risks framework (cumulative incidence analysis). The risk of failing from natural death in the presence of a competing event (sacrifice due to experimental design) (crude cumulative incidence) was evaluated by the method proposed by Prentice. Crude cumulative incidence curves were compared by Gray test [44], which is an extension of log-rank test. To this end, competing risk analysis was performed using the statistical software R with the add-on package “cmprsk” (CumIncidence function), as described by [45].

## CONCLUSIONS

The present study underlines the uniqueness of female physiology and the relevance of ovarian function for longevity. Additionally, these findings underscore the necessity of rigorous studies on sexual dimorphisms for the generation of pharmacological and hormonal therapies that are tailored to female patho-physiology.

## SUPPLEMENTARY FIGURES


