# Supplementary material for: Ovariectomy shortens the life span of female mice
Source: Oncotarget. 2015 Feb 12;6(13):10801–11. doi: 10.18632/oncotarget.2984 (PMC4484420; doi:10.18632/oncotarget.2984)
Supplement: Supplementary file 1 [file oncotarget-06-10801-s001.pdf]

SUPPLEMENTARY FIGURES

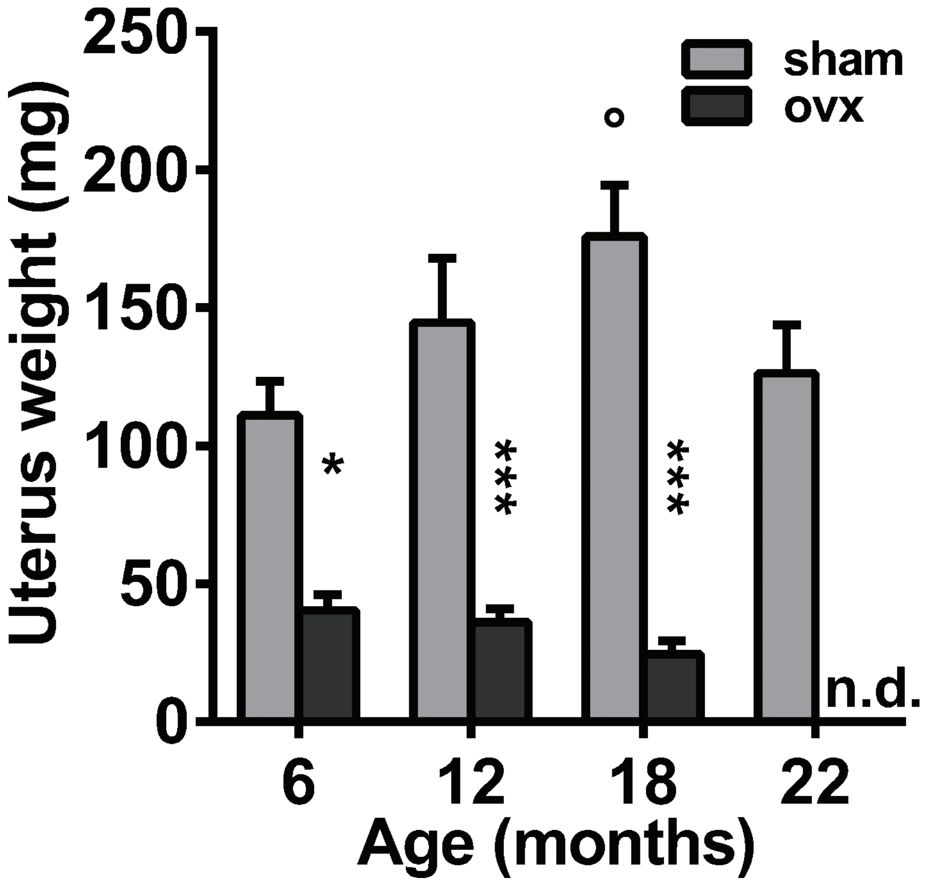

Supplementary Figure S1: Uterus weight in sham operated and ovx female mice at different ages. The uterus weight of each PPRE-lucRepTOP™ mouse was measured before tissue collection. Data are expressed as the mean ± SEM and were analyzed by two-way ANOVA, followed by Bonferroni's *post hoc* test. *$P < 0.05$; **$P < 0.01$; and ***$P < 0.001$. Superimposable data were obtained in the aging experiment using female ERE-LucRepTOP™.

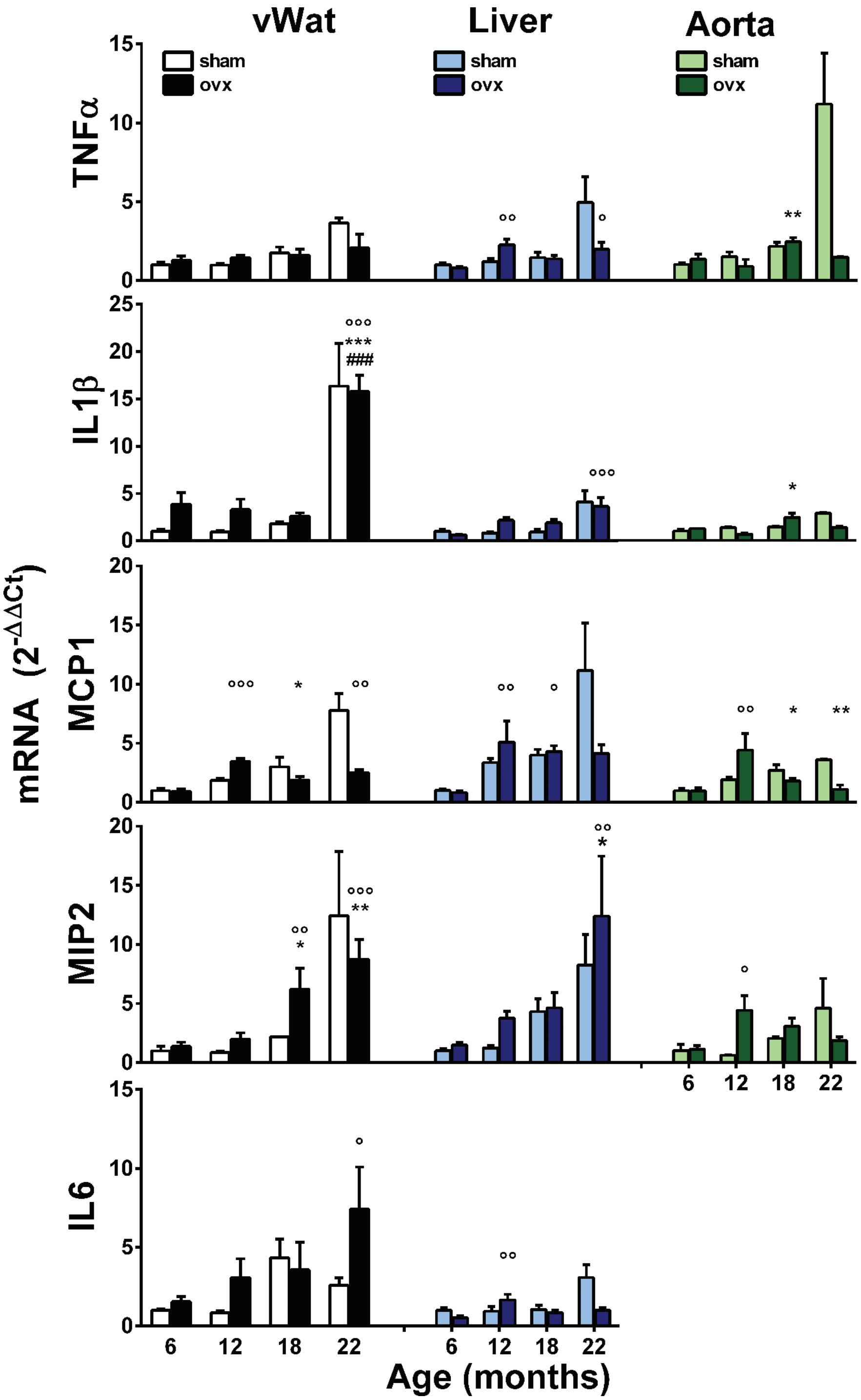

Supplementary Figure S2: Comparative analysis of inflammatory mRNA content in the vWAT, liver and aorta of sham and ovx mice. TNFα, IL1β, MCP-1, MIP-2 and IL-6 mRNA contents were measured by rtPCR using the vWAT, liver and aorta tissue extracts from sham-operated and OVX female mice. Data are expressed as the mean ± SEM. The effect of ovx on the expression of aging-related inflammatory mRNAs was analyzed using one-way ANOVA, followed by Bonferroni's *post hoc* test. °$P < 0.05$; °°$P < 0.01$, °°°$P < 0.001$ *vs*. 6 months; *$P < 0.05$; **$P < 0.01$; and ***$P < 0.001$ *vs*. 12 months; $^{\#}P < 0.05$; $^{\#\#}P < 0.01$, $^{\#\#\#}P < 0.001$ *vs*. 18 months. Unpaired *t*-tests were performed to evaluate the effect of ovariectomy in each age group (Figure 2b). °$P < 0.05$; °°$P < 0.01$; and °°°$P < 0.001$ ovx *vss* sham.

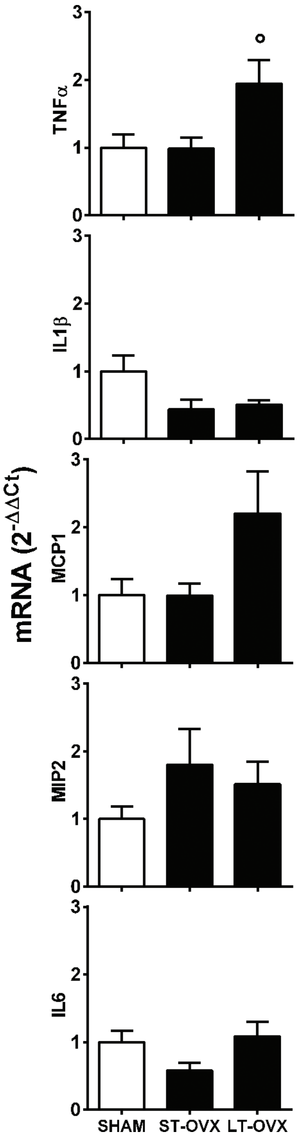

Supplementary Figure S3: Effect of short- and long-term ovx on the production of inflammatory molecules in young mice. TNFα, IL1β, MCP-1, MIP-2 and IL-6 mRNA contents were measured using rtPCR using liver tissue extracts from sham-operated female mice and female mice after OVX for 1 month (ST-OVX) or 4 months (LT-OVX). Data are expressed as the mean ± SEM and were analyzed using one-way ANOVA, followed by Bonferroni's *post hoc* test. $*P < 0.05$; $**P < 0.01$; and $***P < 0.001$.

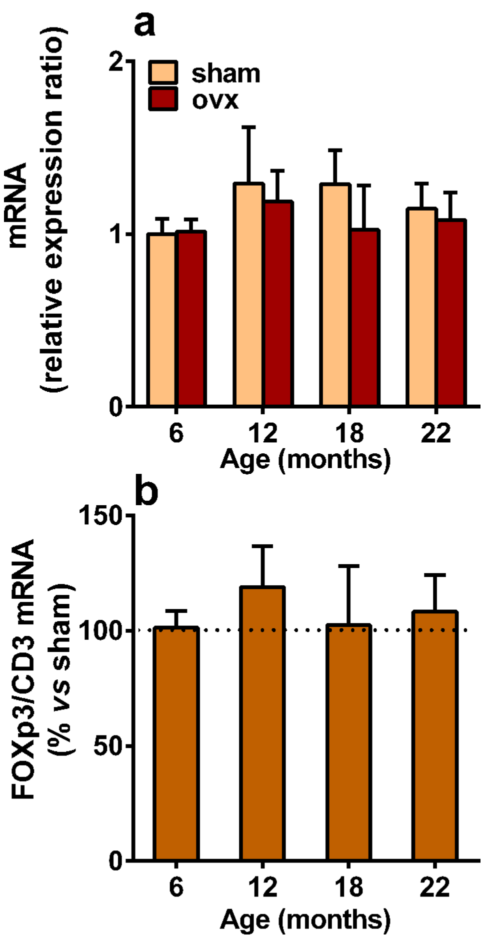

Supplementary Figure S4: Effect of aging and OVX on Foxp3 mRNA content in mouse spleen. (a) Treg activation in the spleen of sham-operated and ovx female mice was expressed as the ratio of Foxp3 to CD3 mRNA content. Data are expressed as the mean ± SEM and were analyzed by two-way ANOVA, followed by Bonferroni's *post hoc* test. **(b)** The Foxp3/CD3 ratio of ovx mice is represented as a percentage of the mean of sham-operated mice of the same age. Data are expressed as the mean ± SEM and were analyzed by one-way ANOVA, followed by Bonferroni's *post hoc* test.
